# Outliving and outlasting: explaining sex inequities in healthy life expectancy through the lens of multimorbidity and within morbidities in older populations in India

**DOI:** 10.3389/fpubh.2025.1613516

**Published:** 2025-12-02

**Authors:** Ajay Kumar

**Affiliations:** Department of Biostatistics and Epidemiology, International Institute for Population Sciences (IIPS), Mumbai, India

**Keywords:** healthy life expectancy, healthy lifespan inequality, multimorbidity, complex multimorbidity, sex disparities, chronic diseases, India

## Abstract

**Background:**

Life expectancy (LE) at birth in India has increased substantially over the past century, initially driven by reductions in infant and childhood mortality and later supported by declining mortality in older population. Despite these gains, sex gaps in LE have widened, and healthy life expectancy (HLE) has declined, largely due to escalating non-communicable diseases (NCDs) and, more recently, a growing burden of multimorbidity among older adults.

**Data:**

Using the Sample Registration System (SRS) and nationally representative Longitudinal Ageing Study in India (LASI) Wave 1, we assessed sex-specific patterns in healthy lifespan inequality (HLI), decomposed disparities in HLE, and quantified inequality arising from mortality and the prevalence of multimorbidity/complex multimorbidity.

**Results:**

Females have a marginal advantage in HLE (30.59 years) compared to males (29.15 years) and have higher multimorbidity-free life expectancy (25.50 years vs. 23.30 years). However, they also live longer with multimorbidity (6.09 years vs. 5.85 years) and spend significantly more time with complex multimorbidity (10.77 years vs. 8.93 years). Mortality differentials remain the dominant contributor to sex-specific HLE gaps. Disease- and multimorbidity-specific patterns reveal a survival–morbidity paradox: women live longer than men but accumulate a greater burden of diseases and conditions. Moreover, cardiovascular and musculoskeletal diseases account for significant losses in disease-free life expectancy (DFLE) among women, while men experience steeper declines due to respiratory diseases.

**Conclusion:**

As India undergoes demographic ageing and an epidemiological shift toward chronic disease dominance, reducing sex disparities in HLE requires targeted public health strategies. Prioritizing prevention, early detection, and integrated management of multimorbidity particularly complex multimorbidity among older women will be essential to narrow the growing gap and promote healthier ageing.

## 1 Introduction

Low-and middle-income countries (LMICs) have experienced steady improvements in life expectancy (LE), now averaging around 70–73 years (1, 2). However, these gains have not been paralleled by comparable increases in healthy life expectancy (HLE), which remains 6–10 years lower, primarily due to the rising burden of non-communicable diseases (NCDs), disabilities, and uneven access to healthcare services (3, 4). India, while sharing this broader LMICs trend, presents a distinct and compelling case for focused research. In 2022, India’s LE was approximately 70.8 years, whereas its HLE (or HALE-Healthy Adjusted Life Expectancy) was only 62.6 years, implying that older adults spend over 8 years in ill health (5). Yet, India is not merely following the LMICs trajectory; it is diverging in significant ways (6). Unlike several LMICs, where infectious diseases still dominate, India is undergoing a rapid epidemiological transition marked by a dual burden: persistent infectious diseases alongside a surge in multimorbidity and age-related chronic conditions (7). This is driven by its fast-ageing population, profound lifestyle changes, and entrenched socio-economic and sex inequalities.

Although India has witnessed remarkable improvements in LE over the past decades, reflecting substantial advancements in healthcare, living standards, nutrition, and public health initiatives (8). Since independence in 1947, LE at birth has risen from approximately 32 years to nearly 70.8 years by 2022 (5). This progress has been largely attributed to reductions in infant mortality, improved maternal health, and the successful control of infectious diseases (9, 10). Key programmes, such as the National Health Mission, have played a pivotal role in extending healthcare access, particularly in rural areas (11–13). Although both males and females have benefited from the declining mortality rates and targeted health interventions. However, the sex gap in LE has fluctuated over these periods and currently stands at 2.7 years, favouring females (14). This disparity or inequality (gap) becomes more pronounced in HLE (15), where sociocultural factors, regional disparities shaped the gap between male and female (16), healthcare accessibility (17), and largely by varying burdens of chronic disease such that certain conditions disproportionately affect younger males, while others contribute more significantly to morbidity in older females (18).

Parallelly, since 1990, India has been undergoing the late phase of epidemiological transition and since recent decades, this transition has been coupled with the rapidly ageing population, underscoring the dual challenges of longevity and changing age pattern of mortality (19–21). This aligns with the ongoing “expansion of morbidity” phase, wherein rising LE is accompanied by a growing proportion of life lived with illness, disability, and poor health (6) and further compounded in recent decades by the escalating burden of multimorbidity, defined as the coexistence of two or more chronic conditions in an individual (22). Moreover, as LE increases, India is facing the ongoing challenge of an ageing population (aged 60+), which is growing in size along with the rising burden of NCDs (23, 24).

In concordance with the above patterns, as the escalating burden of multimorbidity is emerging as a significant public health concern in India (25, 26), particularly among the older population, research has increasingly focused on its association with functional decline, reduced quality of life, elevated mortality risk, decreased healthcare utilisation, and escalating healthcare costs (27–29). Also, prior studies in India estimated that the prevalence of multimorbidity among older adult’s ranges from 23 to 42% (25). Hypertension-diabetes and hypertension-obesity were identified as the most common comorbidity dyads (30). Amid this multimorbidity pattern, in the recent study, the gap in prevalence and risk between males and females was sequentially observed in the rapidly ageing demographics (31).

However, this ripple effects of multimorbidity are not merely semantic; complex multimorbidity captures higher clinical severity and systemic burden by accounting for conditions affecting more than two organ systems (32). While the term “multimorbidity” captures disease count, a standard metric in ageing and health research, this binary threshold often fails to capture the clinical heterogeneity and complexity of chronic disease clustering, and it overlooks the heterogeneity and systemic distribution of conditions. By contrast, complex multimorbidity reflects a higher level of physiological burden and clinical management challenges. International literature emphasises that individuals with complex multimorbidity are more vulnerable to functional limitations, care fragmentation, and polypharmacy, and often require more intensive and coordinated healthcare (32). From a public health standpoint, this operational distinction is important for identifying high-need subgroups, improving care targeting, and understanding differential impacts on population health metrics like HLE. Yet, despite its conceptual and clinical importance, complex multimorbidity remains underutilised in Indian ageing research. Therefore, our study adopts this distinction to provide a more nuanced assessment of multimorbidity’s impact on HLE and its inequality (gap). In doing so, it addresses a crucial empirical gap while aligning with emerging global practice.

Although HLE is a crucial indicator of healthy longevity and obscures substantial variations in healthy lifespan inequality (HLI). HLI reflects the uncertainty surrounding the timing and onset of morbidity, which profoundly influences life-course decisions (33). From a public health perspective, a higher HLI suggests an increase in societal vulnerability and the inefficacy of policies to mitigate life-course risks (34). Despite its importance, evidence on addressing stark inequalities in HLE between older males and females in India, especially in the context of multimorbidity and complex multimorbidity, is limited. While previous studies have explored sex disparities in LE by cause of death (17, 35, 36) research on age-specific HLE inequalities due to multimorbidity is scarce. Thus, it is among the few countries where rapid ageing, uneven healthcare access, and rising multimorbidity intersect so sharply, making it an important case to investigate how multimorbidity influences healthy lifespan and the inequalities.

This study aims to bridge these critical knowledge gaps by providing the first comprehensive analysis of HLE patterns and inequalities related to multimorbidity among India’s older adult population. Our research objectives are threefold: (1) to estimate age-specific HLE for individual morbidities, multimorbidity, and complex multimorbidity among older adults in India; (2) to quantify the causes and magnitude of age-specific gaps in HLE and mortality between males and females for different morbidity patterns; and (3) to assess and quantify the age-specific contribution of complex multimorbidity disparities to overall HLE inequalities (gap) by sex. By focusing on these analytical objectives, this study contributes to the theoretical understanding of health inequality in ageing populations and offers empirical evidence for designing gender-responsive and equity-oriented health interventions in India and similar transitioning LMICs.

## 2 Methods and materials

### 2.1 Mortality data

We used the national representative 2020 Sample Registration System (SRS) statistical report for age-specific mortality rates and construction of abridged life tables. The SRS is a national representative, large-scale demographic survey system designed to provide reliable and robust annual estimates of vital statistics. Mortality rates were extracted for the 45 to 85+year age group. Eight of these groups were defined in five-year intervals, while the final category (85+) represented an open-ended age group.

### 2.2 Morbidities and multimorbidity data

For the age-specific prevalence estimates and computation of HLE for individuals’ morbidities, multimorbidity and complex multimorbidity, we used the national representative Longitudinal Ageing Study in India (LASI, Wave 1, 2017–18) dataset that provides comprehensive details on an extensive number of morbidities for individuals 45 years and older, covering 28 states and 8 UTs of India. LASI is a longitudinal survey that adopted a multi-stage, stratified area probability cluster sampling design, with three stages in rural areas and four stages in urban areas, respectively. The original LASI dataset contained 73,396 individuals. After excluding cases with missing or incomplete health information, our analytical sample comprised 59,830 individuals aged 45 years and older, ensuring robust statistical power for age- and sex-stratified analyses.

### 2.3 Data availability and ethical consideration

Prior informed consent (written and verbal) from all the participants was collected by the field survey agencies. LASI administered consent forms at the household and individual levels, following the Human Subject Protection. The Indian Council of Medical Research (ICMR) extended the necessary guidance and mandatory ethical approval for conducting the LASI survey. The Institutional Ethical Review Board (IRB) at the International Institute for Population Sciences (IIPS) provided additional approval for the study protocol. All methods were carried out under relevant guidelines and regulations by the ICMR.

### 2.4 Measures

#### 2.4.1 Morbidities

We analysed 14 major diseases and conditions using the information on all self-reported and diagnosed diseases available in LASI, which were documented based on responses to the questions, *“Has any health professional ever diagnosed you with the following chronic conditions or diseases?”* and *“In the past 2 years, have you had any of the following diseases?.”* These morbidities were classified according to the International Classification of Diseases, 10th Revision (ICD-10) and included: [(I) Infections; (II) Neoplasms; (III) Blood Diseases; (IV) Endocrine Diseases; (V) Mental and Behavioural Disorders; (VI) Diseases of the Nervous System; (VII) Eye Disorders; (VIII) Ear Diseases; (IX) Cardiovascular Diseases (CVDs); (X) Respiratory System Diseases/Chronic Lung Diseases (CLD); (XI) Digestive System Diseases; (XII) Skin Diseases; (XIII) Musculoskeletal System and Connective Tissue Diseases; and (XIV) Genitourinary System Diseases]. Additional details on morbidity categorisation can be found in Supplementary Table S1.

#### 2.4.2 Multimorbidity and complex multimorbidity

We calculated the age-specific prevalence rate of these 14 diseases and conditions based on the responses and diagnoses available in the LASI survey. Further, a new variable was created and categorised in binary form as: Multimorbidity (Individuals who had combinations of only two diseases/conditions); Complex Multimorbidity (Individuals who had combinations of three or more diseases/conditions).

### 2.5 Statistical analysis

#### 2.5.1 Construction of abridged life table

First, we computed the weighted (sampling weight) age-specific prevalence rates of individual diseases/conditions, multimorbidity and complex multimorbidity to assess their respective burden among older adults in India. Given as,

$$\tau_{x}^{i}=\frac{\text{Population with the morbidity}\;\mathrm{at}\;a\;\text{specified}\;\mathrm{age}}{\text{Population}\;\mathrm{at}\;\text{risk}\;\mathrm{at}\;\text{the specified}\;\mathrm{age}}\times 100$$

Second, the Sullivan’s method was used for constructing the abridged life table from the 45–49 years and above age groups by sex (37). Further, Diseases-Free Life Expectancy (DFLE) for individual diseases/conditions, Multimorbidity-Free Life Expectancy (MFLE) for multimorbidity and Complex Multimorbidity-Free Life Expectancy (CMFLE) for complex multimorbidity were calculated using the life table. The columns of the abridged life table were constructed using standard demographic formulas, as outlined below:

_n_M*_x_* = central death rate in interval *x* to *x* + n

$$mn_{x}=\frac{dn_{x}}{Ln_{x}}$$

_n_q*_x_*: probability of dying between age *x* to *x* + n

$$qn_{x}=\frac{n.mn_{x}}{1+(n-an_{x}).mn_{x}}$$

$l_{x}$ : number of people alive at exact age x , among a hypothetical birth cohort of 100,000, called the radix of the life table

$l_{x+n}$ : number of people alive at the exact age x + n

$$l_{x+n}=l_{x}.(1-\mathrm{qnx})$$

_n_𝑑_𝑥_: number of deaths in the age interval 𝑥 to 𝑥 + n

$${\mathrm{dn}}_{x}=l_{x}.nq_{x}$$

_n_𝑎_𝑥_: average number of years lived in the age interval 𝑥 to 𝑥 + n

$$an_{x}=\frac{Ln_{x}-n.l_{x+n}}{l_{x}-l_{x+n}}$$

_n_𝐿_𝑥_: total number of person–years lived between exact ages 𝑥 to 𝑥 + 𝑛

$$Ln_{x}=n.l_{x+n}+a_{x}.dn_{x}$$

T_𝑥_: total number of person–years lived above age 𝑥

$$T_{x}=T_{x+n}+Ln_{x}$$

e_𝑥_: average number of years of life remaining for a person alive at the beginning of age interval 𝑥

$$e_{x}=\frac{T_{x}}{l_{x}}$$

Further, the following formulas were used to calculate healthy life expectancy:

$$Ln_{x}^{H}=\text{Healthy}\;\;\text{person}-\text{years}$$

$$Ln_{x}^{H}=Ln_{x}.(1-\tau_{x}^{i})$$

$\tau_{x}^{i}$ : age-specific prevalence (proportion unhealthy) in that interval.

Total healthy person-years above age x

$$T_{x}^{H}=\sum_{y\geq x}Ln_{y}^{H}$$

Healthy Life Expectancy (HLE)

$$e_{x}^{H}=\frac{T_{x}^{H}}{l_{x}}$$

Finally, the distributional composition of unhealthy years lived by individuals due to multimorbidity and complex multimorbidity was calculated by subtracting DFLE, MFLE and CMFLE from life expectancy (LE).

#### 2.5.2 Healthy lifespan inequality (gap)

The Decomposition method was used to quantify the contributions of mortality and different causes to inequality in summary health metrics (HLE). We used the step-wise replacement decomposition method to decompose the age-specific sex gap in HLE to mortality and different causes (38). This method assesses the impact of observed differences in parameters (mortality and causes) between two populations (male and female) on the respective indicator (DFLE, MFLE and CMFLE) or HLE (in common), treating these as the non-linear function of these parameters. This decomposition method isolates the effect of each component of a parameter by altering one element at a time and recalculating the aggregate measure. This process estimates the individual contribution of that element to the overall difference between groups. The total contribution of the parameter is then obtained by summing the contributions of all its elements. Thus, the mathematical formula of the step-wise replacement decomposition method used in this study to obtain mortality and causes (multimorbidity and complex multimorbidity) is given.

Mortality Component ( $\alpha_{x}$ ): The mortality component can be expressed in a simplified form to capture the contribution of differences in survival probabilities and life table ratios;

$$\begin{array}{l} \alpha_{x}=(\frac{l_{x}^{1}+l_{x}^{2}}{2})(\theta_{x}^{1}-\theta_{x}^{2})\;(\frac{\tau_{x}^{1}+\tau_{x}^{2}}{2})\\ +(\frac{\frac{\mathrm{DFL}E_{x+1}^{1}}{l_{x}^{2}}+\frac{\mathrm{DFL}E_{x+1}^{2}}{l_{x}^{1}}}{2})(q_{x}^{1}-q_{x}^{2}). \end{array}$$

Where:

$l_{x}^{i}\;$ = Survivors at age x in population i (sex 1 = male, 2 = female).

$\theta_{x}^{i}\;$ = $L_{x}^{i}/l_{x}^{i}$ (Person-year lived per survivor).

$\tau_{x}^{i}\;$ = Morbidity prevalence.

$q_{x}^{i}\;$ = Probability of dying at age x.

Causes Component ( $\beta_{x}$ ): the causes component reflects the contribution of differences in healthy proportions:

$$\beta_{x}=(\frac{l_{x}^{1}+l_{x}^{2}}{2})\;(\theta_{x}^{1}-\theta_{x}^{2})\;(\frac{\tau_{x}^{1}+\tau_{x}^{2}}{2})$$

Overall HLE gap: the total gap in HLE between the two populations (males and females) was obtained by summing the contributions of the mortality and morbidity components over all age groups.

$$\mathrm{HL}E_{x}^{2}-\mathrm{HL}E_{x}^{1}=\sum_{j=x\;\;}^{z}\alpha_{j\;\;\;}+\sum_{j=x\;\;}^{z}\beta_{j\;\;\;}$$

Where:

$\alpha_{j}$ : Contribution from mortality differences at age j

$\beta_{j}$ : Contribution from causes differences at age j

z : Final age group (85 + years)

**Figure 1:**
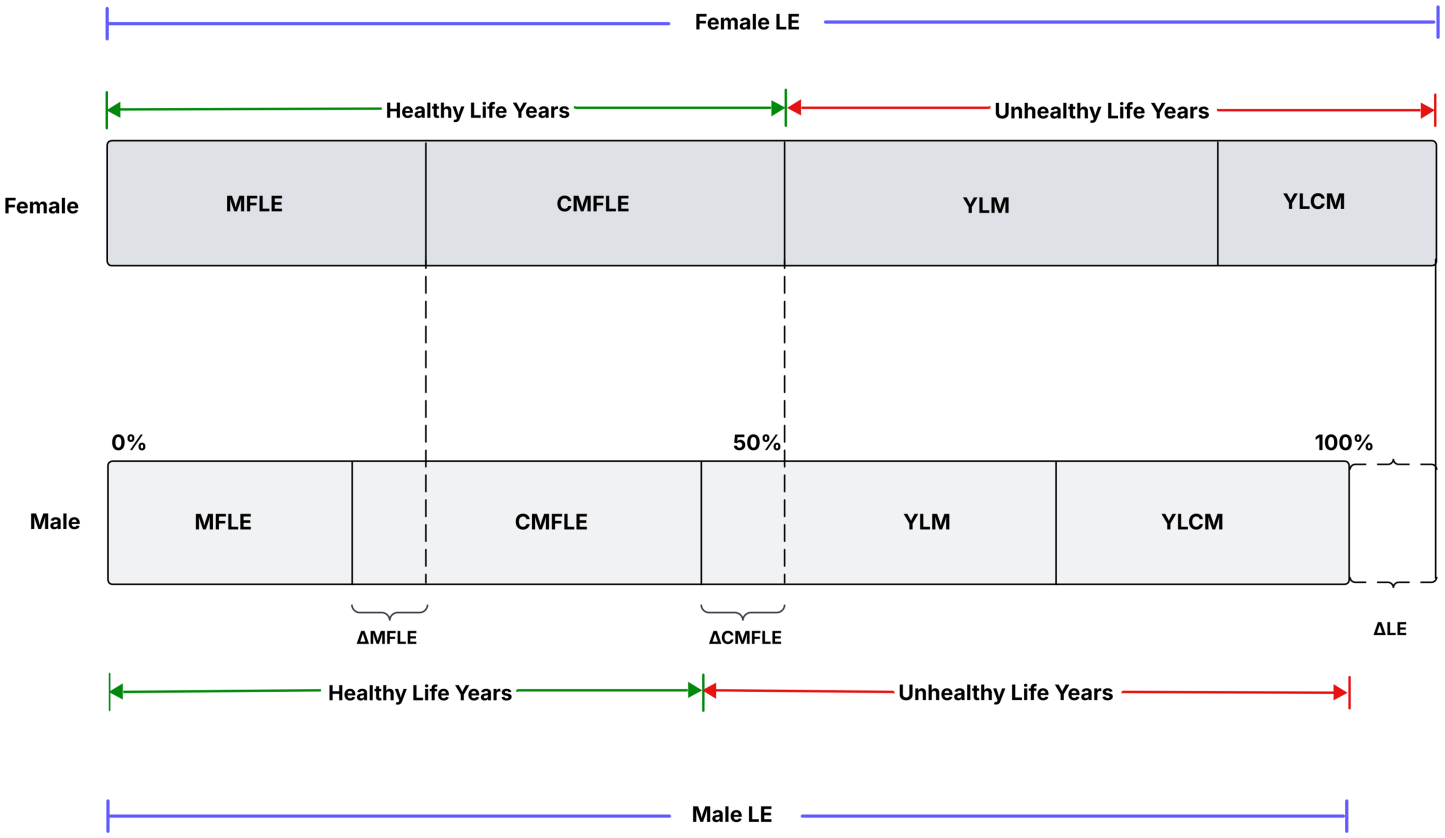
Hypothetical and systematic representation of male–female inequality (gap) in HLE, LASI Wave-1 (2017−18), India.

All analyses were performed with R Studio version 2024.12.1–563^1^ and STATA 17. Further, Figure 1 presents a proportional breakdown of LE into healthy and unhealthy years, further divided into Multimorbidity-Free Life Expectancy (MFLE), Complex Multimorbidity-Free Life Expectancy (CMFLE), Years Lived with Multimorbidity (YLM), and Years Lived with Complex Multimorbidity (YLCM) for males and females.

## 3 Results

### 3.1 Age-specific life expectancy and healthy life expectancy patterns

Table 1 depicts the estimates of LE and healthy life expectancy (HLE) as multimorbidity-free life expectancy (MFLE), and complex multimorbidity-free life expectancy (CMFLE) for males and females in addition to the age-specific sex gap. The table highlights distinct patterns in MFLE and CMFLE between males and females across age groups. LE declines with age for both sexes, but females continue to maintain a higher LE across all age groups. At ages 45–49, females have an LE of 31.59 years compared to 29.15 years for males. However, female also experience a higher MFLE (25.50 years vs. 23.30 years) and slightly higher CMFLE (20.82 years vs. 20.22 years), although a prevalence gap of 3.53% was observed in complex multimorbidity (Female 33.25% vs. Male 29.72%) than the gap of 0.35% in multimorbidity favouring male (Female 19.63% vs. Male 19.98%; see Table 2). This table indicates that females not only live longer but also remain free of multimorbidity for a slightly greater number of years than males in younger years; however, the age-specific gap in complex multimorbidity is scuttled but constant (0.61 years in the 45–49 age-group to 0.18 years in the 85+). Although the gap in MFLE and CMFLE is more pronounced in younger age groups, but diminishes in later older populations, with near parity by 85+. As the population ages, the advantage of females remaining free of both multimorbidity and complex multimorbidity diminishes.

**Table 1:** Age-specific estimates of life expectancy (LE), multimorbidity-free life expectancy (MFLE), and complex multimorbidity-free life expectancy (CMFLE) for males and females and inequality (gap/difference), Pooled refers to as Individuals who had combinations of two or more diseases/conditions, LASI Wave-1 (2017−18), India.

Age	Male				Female				Inequality (Gap)			
	LE	MFLE	CMFLE	Pooled	LE	MFLE	CMFLE	Pooled	Δ LE	Δ MFLE	Δ CMFLE	Δ Pooled
45–49	29.15	23.30	20.22	14.36	31.59	25.50	20.83	14.73	2.44	2.20	0.61	0.37
50–54	24.99	19.84	16.67	11.52	27.13	21.88	17.35	12.09	2.15	2.04	0.68	0.57
55–59	21.02	16.71	13.50	9.18	22.96	18.50	14.21	9.75	1.94	1.79	0.71	0.57
60–64	17.54	14.01	10.96	7.42	19.13	15.40	11.53	7.79	1.59	1.39	0.57	0.37
65–69	14.26	11.39	8.54	5.67	15.52	12.47	9.01	5.96	1.25	1.08	0.47	0.29
70–74	11.43	9.15	6.66	4.38	12.35	9.98	6.94	4.57	0.92	0.83	0.28	0.19
75–79	9.04	7.24	5.07	3.26	9.62	7.72	5.40	3.49	0.58	0.48	0.33	0.23
80–84	6.96	5.57	3.72	2.33	7.23	5.96	3.98	2.70	0.27	0.39	0.26	0.37
85+	5.42	4.38	2.91	1.86	5.42	4.32	3.09	1.99	0.00	−0.06	0.18	0.13

**Table 2:** Age-specific prevalence rates of multimorbidity and complex multimorbidity stratified by sex, Pooled refers to as Individuals who had combinations of two or more diseases/conditions, LASI Wave-1 (2017−18), India.

Age group			45–49	50–54	55–59	60–64	65–69	70–74	75–79	80–84	85+	Total % (95% CI)
Multimorbidity	Two	Male	17.51	20.96	21.82	20.05	20.7	19.91	19.83	20.56	19.30	19.98% (19.50–20.47)
		Female	18.88	19.03	19.14	19.12	20.75	18.09	22.4	15.46	20.35	19.63% (19.19–20.08)
Complex Multimorbidity	Three	Male	9.04	10.67	13.12	13.57	14.28	13.59	14.68	19.82	22.22	13.12% (12.74–13.51)
		Female	11.98	12.02	13.09	13.22	12.85	12.65	16.21	20.25	18.57	13.91% (13.53–14.30)
	Four	Male	4.71	7.86	10.32	8.19	10.71	9.87	12.43	11.47	10.99	8.22% (7.93–8.52)
		Female	5.56	7.75	9.05	9.02	12.67	10.12	10.37	11.29	9.85	8.83% (8.52–9.15)
	Five	Male	2.03	2.56	3.31	4.3	6.15	6.79	6.43	7.13	4.17	4.12% (3.89–4.36)
		Female	3.06	3.80	4.31	5.10	5.33	8.44	6.43	4.94	7.03	5.26% (5.02–5.50)
	Six	Male	0.54	1.18	1.67	2.13	2.36	4.04	3.77	3.24	4.45	2.16% (1.99–2.34)
		Female	1.64	1.93	1.94	2.82	4.06	4.00	4.25	5.79	3.42	2.77% (2.60–2.94)
	Seven	Male	0.39	0.38	1.06	1.23	1.82	2.35	1.76	3.05	1.98	1.14% (1.02–1.27)
		Female	0.64	0.75	1.75	1.55	1.47	2.15	2.22	2.17	1.76	1.23% (1.11–1.36)
	Eight Plus	Male	0.83	0.41	0.49	1.15	1.40	1.52	2.03	1.89	2.59	0.97% (0.87–1.09)
		Female	0.50	0.55	1.94	1.52	1.21	6.32	3.15	2.15	2.31	1.26% (1.14–1.40)
	Complex Total	Male	17.54	23.06	29.97	30.57	36.71	38.16	41.1	46.6	46.4	29.72% (29.21–30.23)
		Female	23.38	26.8	32.07	33.24	37.58	43.68	42.63	46.58	42.94	33.25% (32.77–33.73)
Pooled	Male		35.05	44.02	51.8	50.63	57.41	58.07	60.93	67.16	65.7	49.71% (49.23–50.19)
	Female		42.26	45.83	51.22	52.36	58.33	61.77	65.02	62.04	63.28	52.88% (52.44–53.33)

**Figure 2:**
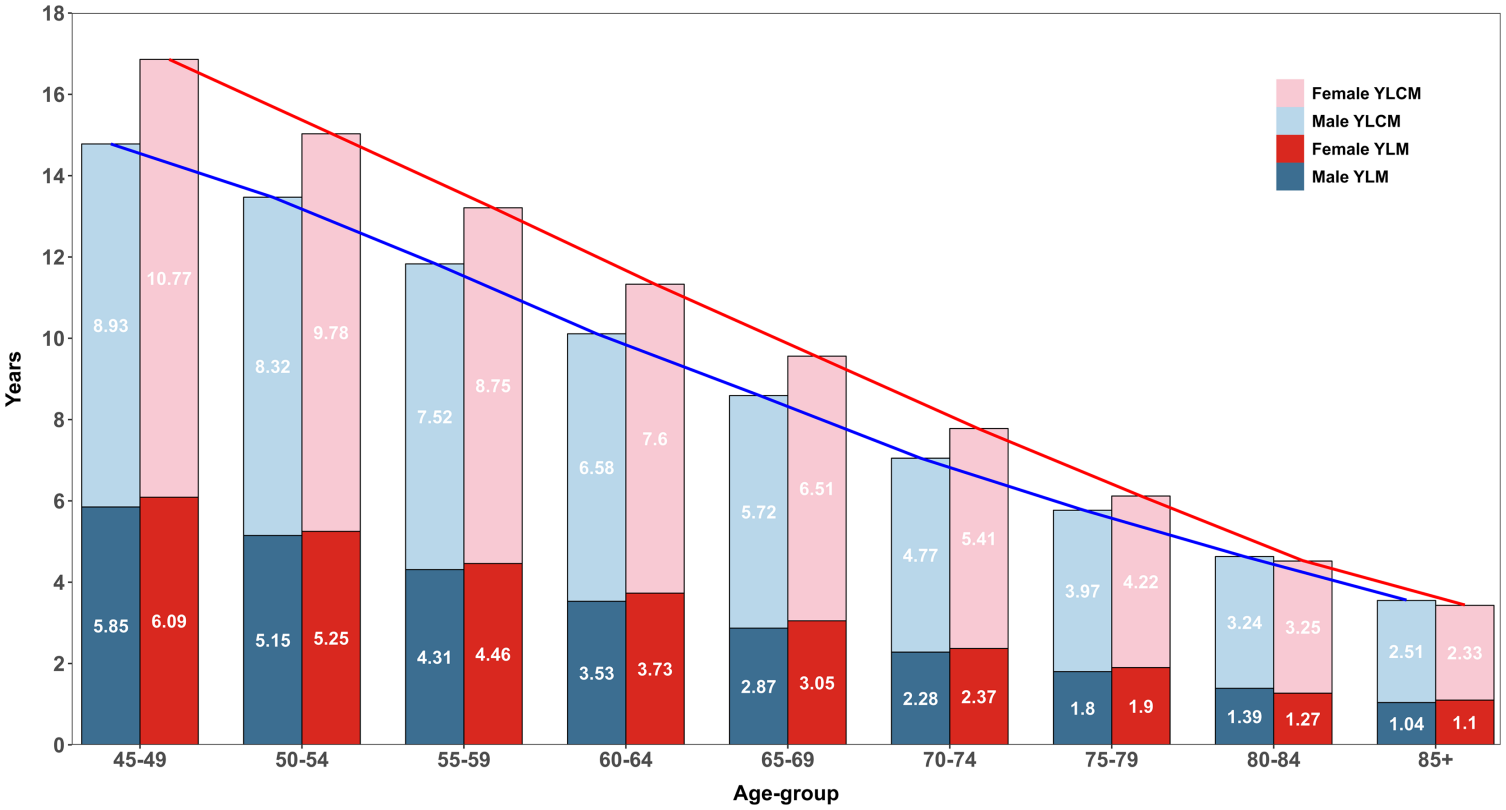
Age-specific estimates of years lived with multimorbidity (YLM) and complex multimorbidity (YLCM) stratified within and between sex, LASI Wave-1 (2017−18), India.

Figure 2 describes the age-specific pattern of years lived with multimorbidity (YLM) and complex multimorbidity (YLCM) for males and females. At ages 45–49, females have an LE of 31.59 years compared to 29.15 years for males (see Table 1), but spend more time with multimorbidity (6.09 years vs. 5.85 years, ∆0.24 years) and significantly higher in complex multimorbidity (10.77 years vs. 8.93 years, ∆1.84 years). This pattern persists across subsequent age groups, with females consistently showing higher YLM and YLCM relative to males. However, the proportion of years lived with these conditions diminishes, particularly in older age groups (e.g., 70–74 and beyond), where a significant share of remaining years were characterised by multimorbidity. Although YLCM in males (blue line) declines more sharply with age compared to females, who experience a slower reduction rate, this suggests that females not only live longer but also endure a prolonged period of life with ill/poor health. Moreover, it was clearly observed that in total (red line), females spend more time in overall bad health conditions in each age group and the disparity reduced in later life courses.

### 3.2 Decomposition

#### 3.2.1 Age-specific contribution of mortality and multimorbidity/complex multimorbidity in HLE gap

**Figure 3:**
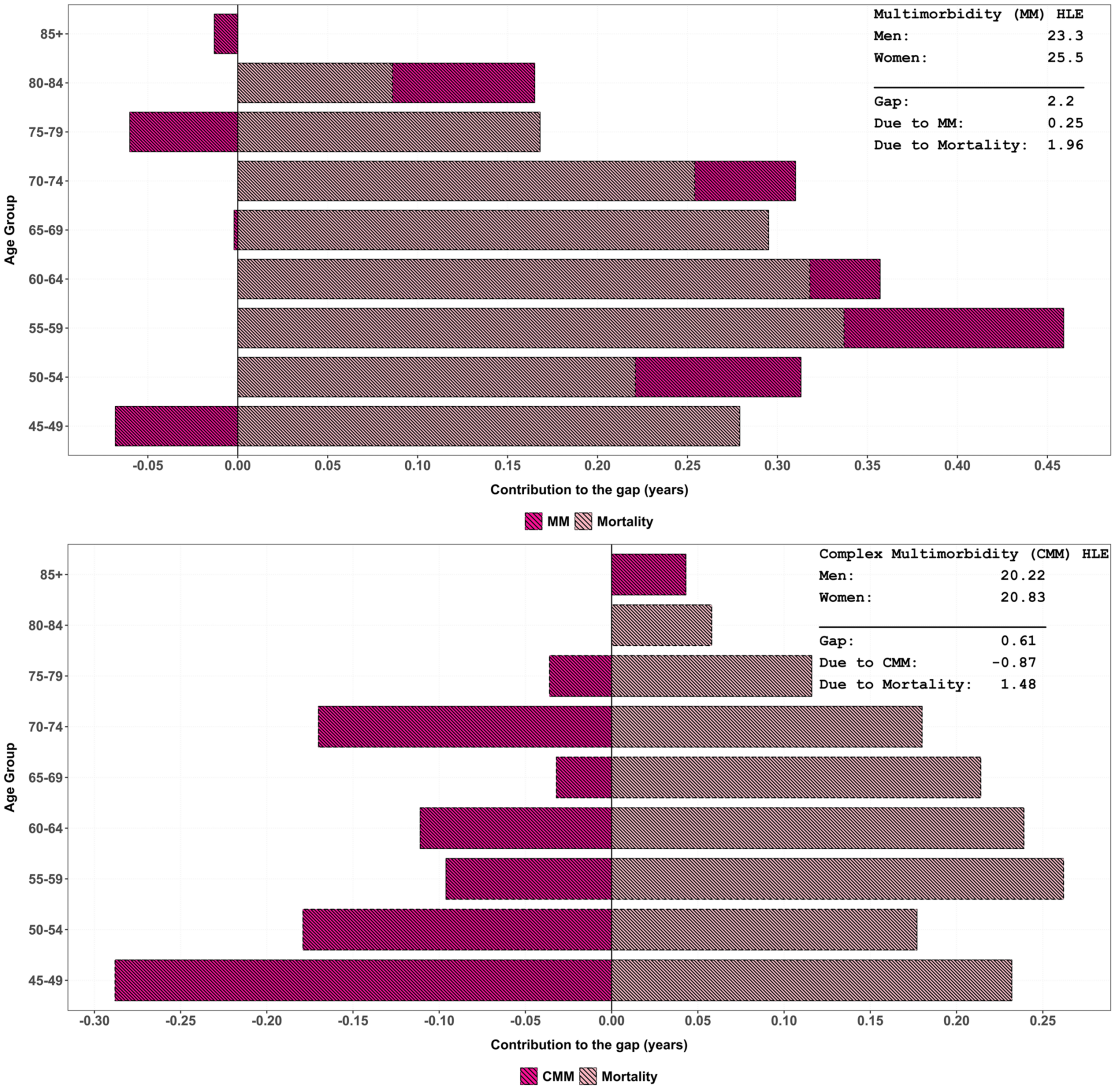
Age-specific contribution of multimorbidity/complex multimorbidity and mortality to change in HLE inequality by sex, LASI Wave-1 (2017−18), India.

Table 3 depicts the age-specific contribution of multimorbidity/complex multimorbidity and mortality between males and females in HLE. Negative values indicated that cause-specific differences might mitigate mortality-driven gaps and reflected shared disease patterns and rates. The gap of multimorbidity in HLE was 2.20 years, with mortality differences accounting for 1.96 years and multimorbidity contributing 0.25 years (also see Figure 3). As multimorbidity complexity increases, mortality remains the dominant driver of HLE gaps across all age-groups, with contributions ranging from 2.06 years (three conditions) to the highest gap of 2.40 years (seven conditions). In contrast, complex-multimorbidity specific contributions are minimal or negative, such as −0.11 years for three conditions and −0.24 years for eight plus conditions. Further, Figure 3 also suggests that the contribution due to differences in overall complex multimorbidity is much higher and negative than that due to differences in only multimorbidity.

**Table 3:** Age-specific and total inequality (gap) in healthy life expectancy (HLE) due to multimorbidity/complex multimorbidity, and the extent to which this gap is explained by multimorbidity/complex multimorbidity and mortality between males and females, LASI Wave 1 (2017–18), India.

Causes	Morbidity counts	45–49	50–54	55–59	60–64	65–69	70–74	75–79	80–84	85+	Gap explained by causes	Gap explained by mortality	Total gap in HLE
Multimorbidity	Two	−0.07	0.09	0.12	0.04	0.00	0.06	−0.06	0.08	−0.01	0.25	1.96	2.20
Complex multimorbidity	Three	−0.15	−0.07	0.00	0.02	0.05	0.03	−0.04	−0.01	0.05	−0.11	2.06	1.95
	Four	−0.04	0.01	0.06	−0.04	−0.07	−0.01	0.05	0.00	0.01	−0.03	2.19	2.16
	Five	−0.05	−0.06	−0.05	−0.03	0.03	−0.05	0.00	0.03	−0.04	−0.21	2.29	2.08
	Six	−0.05	−0.04	−0.01	−0.03	−0.06	0.00	−0.01	−0.04	0.01	−0.23	2.35	2.12
	Seven	−0.01	−0.02	−0.03	−0.01	0.01	0.01	−0.01	0.01	0.00	−0.05	2.40	2.35
	Eight Plus	0.02	−0.01	−0.07	−0.02	0.01	−0.15	−0.03	0.00	0.00	−0.24	2.39	2.15
	Complex Total	−0.29	−0.18	−0.10	−0.11	−0.03	−0.17	−0.04	0.00	0.04	−0.87	1.48	0.61
Pooled		−0.36	−0.09	0.03	−0.07	−0.03	−0.11	−0.10	0.08	0.03	−0.63	1.00	0.37

The total HLE gap within complex multimorbidity narrows slightly as the number of conditions increases, from 2.16 years for four conditions to 2.15 years for eight plus. When considering combined complex multimorbidity, the net effect comprised a gain of 1.47 years due to higher mortality among males and a loss of 0.87 years due to higher morbidity rates among females. Although the gap differential of −0.87 years is explained by total complex multimorbidity, it is the highest among all causes. For all-cause gaps, the total gap further reduces to 0.37 years, primarily driven by mortality disparities (1.00 years). These findings emphasise the critical role of mortality in shaping HLE gaps, especially in elderly populations with complex multimorbidity.

#### 3.2.2 Age-specific contribution within complex multimorbidity in HLE gap

**Figure 4:**
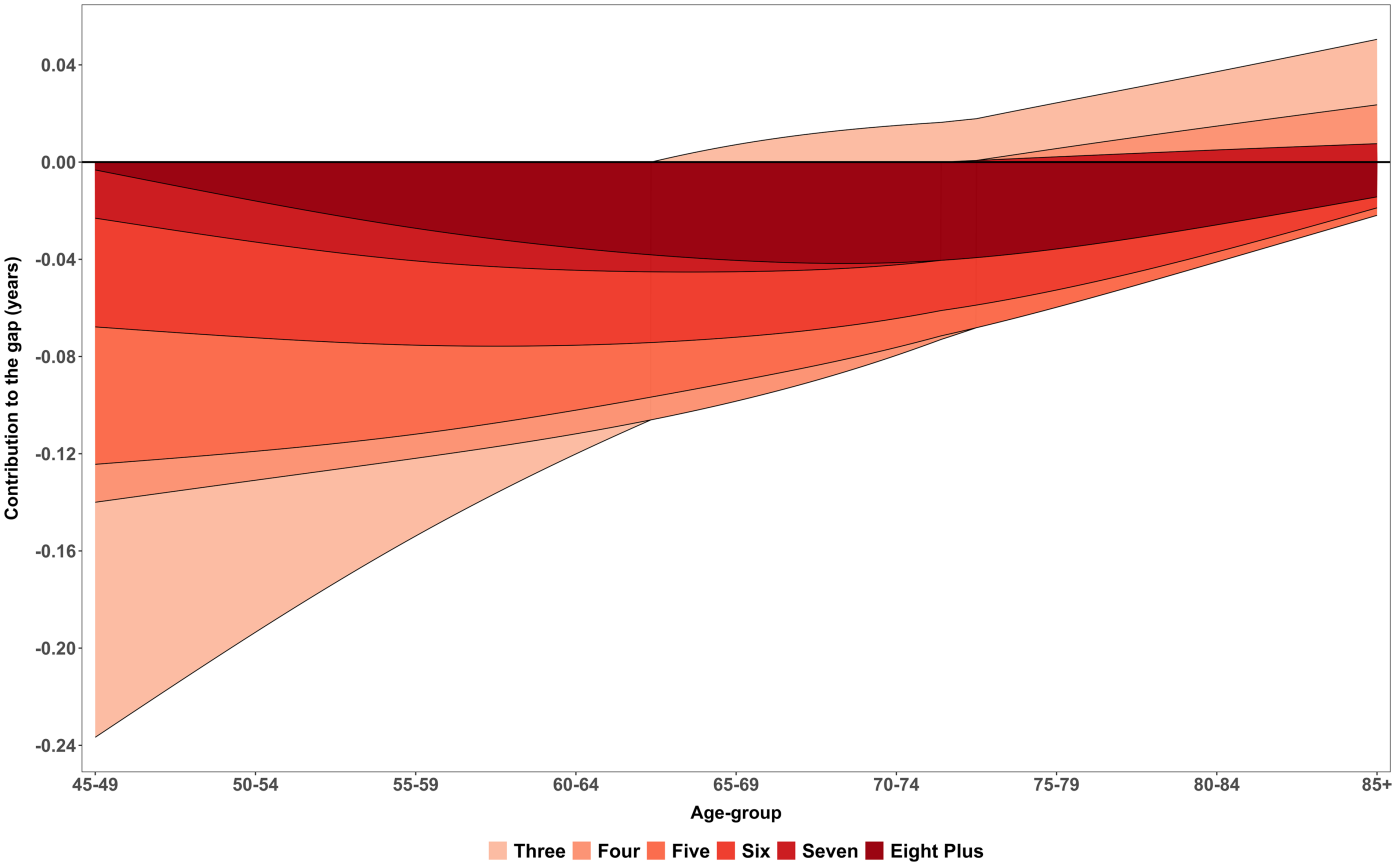
Age-specific contribution (smooth pattern) of morbidity measure within complex multimorbidity to changes in CMFLE inequality (gap) between males and females. LASI Wave-1 (2017−18), India.

Figure 4 illustrates the smooth patterns in age-specific contribution within complex multimorbidity to changes in complex multimorbidity-free life expectancy (CMFLE) inequality between males and females, derived using the stepwise replacement decomposition method (see Table 3 for exact contribution) while the estimates of within-CMFLE are presented in Supplementary Table S2. The figure clearly demonstrates that as the number of morbidity/conditions increases, the negative contribution to the gap in CMFLE between female and male becomes more pronounced. Specifically, female who experience five or more morbidities consistently show the largest reductions in morbidity-free years as compared to men. While a bell-shaped trend in diparity was observed in eight plus morbidities and peaked in middle-aged groups. These reductions begin significantly early in older adulthood, particularly in the age group 45–54, where the contribution to the gap approaches nearly −0.20 years. This pattern indicates a steep decline in healthy life years for women with high multimorbidity burden during midlife relative to their male counterparts. The presence of multiple chronic diseases at this early stage likely plays a critical role in defining subsequent health trajectories and life expectancy differences.

Further inspection across the age spectrum reveals that the negative contributions from higher morbidity levels persist well into older age, stretching up to the 85 + group. Although there is some attenuation of this gap among the oldest age categories, the reductions in CMFLE among women with five, six, or seven chronic conditions remain substantial. This persistence highlights a sustained health disparity that does not fully diminish with age. The continued negative impact across all age groups underscores the long-term burden of complex multimorbidity for women, reaffirming that the inequality in morbidity-free life expectancy between sexes is a pervasive issue throughout the aging process rather than confined to earlier or later life periods.

#### 3.2.3 Age-specific prevalence rates of morbidities and DFLE estimates by sex

Table 4 presents the age and sex-specific prevalence rates of morbidities among males and females aged 45 and above. Eye disorders are the most prevalent conditions, affecting nearly half of both males (47.74%) and females (48.15%) overall, with rates increasing steadily with age, reaching over 66% in the oldest age groups. Cardiovascular diseases (CVDs) represent the second most common morbidity, with a notably higher prevalence in females (31.54%) compared to males (25.49%), especially from age 50 onwards, where rates rise sharply, peaking around 41–42% in females aged 70–84.

**Table 4:** Age-specific prevalence rate of morbidities stratified by sex, LASI Wave-1 (2017−18), India.

Morbidity	Sex	45–49	50–54	55–59	60–64	65–69	70–74	75–79	80–84	85+	Total % (95% CI)
Infections	Male	4.81	4.39	3.55	3.98	3.68	4.28	3.46	1.63	1.04	4.00% (3.78–4.23)
	Female	3.60	3.35	2.81	2.57	2.99	3.60	3.67	1.63	0.76	3.13% (2.95–3.31)
Neoplasms	Male	0.41	0.33	0.34	0.58	0.59	0.38	0.61	2.11	0.19	0.48% (0.41–0.56)
	Female	0.54	0.97	0.73	0.73	0.65	1.24	0.73	0.29	0.81	0.77% (0.68–0.87)
Blood diseases	Male	2.63	2.77	3.41	3.00	3.23	3.72	3.50	2.16	3.96	3.08% (2.89–3.28)
	Female	6.48	5.28	5.56	6.54	5.54	5.32	6.10	9.31	4.77	5.88% (5.64–6.13)
Endocrine diseases	Male	8.49	13.86	16.19	16.29	17.43	17.12	16.45	12.49	17.22	14.63% (14.24–15.03)
	Female	14.02	13.69	21.02	16.87	19.83	20.77	13.11	11.67	9.27	16.34% (15.96–16.73)
Mental and behavioural disorders	Male	1.46	0.84	0.71	1.26	0.77	0.96	1.28	0.96	2.75	1.08% (0.97–1.20)
	Female	0.81	1.03	0.84	0.87	1.33	1.55	2.96	1.63	3.12	1.22% (1.11–1.34)
Diseases of the nervous system	Male	1.78	1.14	1.38	1.55	1.57	1.45	1.78	1.70	2.47	1.53% (1.39–1.67)
	Female	1.43	0.97	1.06	1.25	1.08	1.15	1.95	1.54	1.92	1.24% (1.13–1.36)
Eye disorders and adnexa	Male	37.61	43.32	43.74	46.48	54.34	55.67	57.05	66.98	66.49	47.74% (47.19–48.29)
	Female	38.44	40.25	44.8	48.78	54.96	62.17	63.26	56.41	65.69	48.15% (47.59–48.72)
Ear diseases	Male	3.33	3.84	4.98	5.56	8.18	12.17	13.75	16.06	16.59	6.89% (6.62–7.18)
	Female	3.83	5.00	4.69	5.83	8.78	12.05	10.95	13.13	18.66	6.90% (6.64–7.18)
Cardiovascular diseases (CVDs)	Male	14.75	21.86	25.41	26.17	32.00	32.56	32.55	37.83	29.29	25.49% (24.94–26.05)
	Female	21.98	25.86	31.11	33.77	37.68	41.88	41.83	42.02	36.27	31.54% (30.98–32.10)
Respiratory system diseases/CLD	Male	3.05	4.67	11.17	6.73	9.01	10.73	10.77	9.78	7.82	7.23% (6.94–7.52)
	Female	3.33	4.20	6.62	6.06	5.94	11.30	10.28	10.15	11.24	6.14% (5.88–6.41)
Digestive system diseases	Male	17.46	15.96	18.43	19.53	19.28	19.08	21.08	18.11	16.28	18.26% (17.80–18.73)
	Female	16.01	17.11	18.35	19.35	17.57	17.65	20.66	22.64	15.07	17.75% (17.33–18.18)
Skin diseases	Male	5.05	6.83	5.39	5.52	5.69	6.11	5.53	5.35	9.91	5.88% (5.61–6.15)
	Female	4.25	4.61	5.77	4.84	3.99	4.98	4.08	6.11	5.58	4.66% (4.44–4.89)
Musculoskeletal system and connective tissue diseases	Male	6.66	9.06	14.01	14.7	16.47	15.91	18.13	15.83	16.40	12.77% (12.37–13.18)
	Female	13.12	16.35	18.65	18.92	23.33	27.57	23.82	24.35	21.03	19.08% (18.64–19.53)
Genitourinary system diseases	Male	6.29	5.95	5.80	7.62	8.38	10.54	12.02	12.63	13.36	7.83% (7.52–8.14)
	Female	4.87	4.30	4.87	5.47	5.37	5.94	7.20	9.40	7.03	5.29% (5.05–5.54)

Musculoskeletal system and connective tissue diseases also show significant sex differences, with females exhibiting consistently higher prevalence (19.08%) than males (12.77%), especially after age 50. Endocrine diseases, including diabetes, are more prevalent in females (16.34%) compared to males (14.63%), though male prevalence peaks slightly higher in old age (85+). Blood diseases are similarly more common in females (5.88%) than in males (3.08%). On the other hand, respiratory system diseases or chronic lung diseases are slightly more prevalent in males overall (7.23%) compared to females (6.14%).

Other morbidities such as infections, mental and behavioural disorders, digestive system diseases, and skin diseases, show smaller overall prevalence rates but with varying patterns by age and sex. Notably, infections and blood diseases tend to be more prevalent in females across age groups, whereas infections peak in males between 45 to 50 years and decline thereafter. Digestive system diseases maintain similar levels between sexes, around 18%. Mental and behavioural disorders are relatively low in prevalence overall but slightly higher in females (1.22%) than males (1.08%).

In summary, Table 4 highlights important sex- and age-related variations in morbidity patterns: females bear a heavier burden of CVDs, musculoskeletal and endocrine diseases, while males have a higher prevalence of respiratory diseases and infections at younger ages. These differences warrant sex-specific health interventions and resource planning tailored by age group to effectively address the dominant health challenges in ageing populations.

Table 5 displays the diseases-free life expectancy (DFLE) across age groups, stratified by sex and specific morbidities. Females generally demonstrate longer DFLE compared to males for most diseases, reflecting a consistently higher number of expected years lived free from these conditions. For instance, at ages 45–49, females have notably greater DFLE for infections (30.64 vs. 28.02 years), neoplasms (31.35 vs. 29.00 years), and blood diseases (29.70 vs. 28.25 years). Similar trends extend across endocrine diseases, mental and behavioural disorders, skin diseases, and diseases of the nervous system, where females enjoy more disease-free years, although this advantage tends to narrow with advancing age.

**Table 5:** Age-specific disease-free life expectancy (DFLE) and inequality (gap) due to morbidities between males and females, LASI Wave 1 (2017–18), India.

Morbidity	Sex	45–49	50–54	55–59	60–64	65–69	70–74	75–79	80–84	85+	Gap in years
Infections	Male	28.02	24.07	20.29	16.93	13.79	11.07	8.83	6.86	5.37	2.62
	Female	30.64	26.35	22.32	18.60	15.07	12.00	9.40	7.14	5.38	
Neoplasms	Male	29.00	24.86	20.90	17.42	14.17	11.34	8.96	6.87	5.41	2.35
	Female	31.35	26.92	22.79	18.98	15.39	12.24	9.56	7.20	5.38	
Blood diseases	Male	28.25	24.19	20.33	16.97	13.79	11.04	8.75	6.75	5.21	1.45
	Female	29.70	25.54	21.58	17.95	14.58	11.58	8.97	6.71	5.16	
Endocrine diseases	Male	24.88	21.02	17.58	14.65	11.91	9.59	7.65	5.95	4.49	1.53
	Female	26.41	22.57	18.94	15.96	12.96	10.49	8.49	6.47	4.92	
Mental and behavioural disorders	Male	28.83	24.73	20.80	17.33	14.10	11.28	8.90	6.84	5.27	2.34
	Female	31.18	26.75	22.62	18.81	15.21	12.07	9.37	7.07	5.25	
Diseases of the nervous system	Male	28.70	24.61	20.69	17.25	14.02	11.23	8.87	6.82	5.29	2.49
	Female	31.19	26.80	22.66	18.87	15.29	12.15	9.45	7.11	5.32	
Eye disorders and adnexa	Male	15.05	12.36	10.03	7.92	6.00	4.61	3.42	2.31	1.82	0.82
	Female	15.87	13.06	10.46	8.22	6.23	4.70	3.68	2.85	1.86	
Ear diseases	Male	27.03	22.97	19.11	15.73	12.54	9.83	7.68	5.83	4.52	2.16
	Female	29.19	24.88	20.88	17.17	13.70	10.75	8.32	6.10	4.41	
Cardiovascular diseases (CVDs)	Male	21.60	17.96	14.78	12.11	9.60	7.65	6.03	4.59	3.83	−0.40
	Female	21.21	17.67	14.49	11.78	9.31	7.28	5.72	4.38	3.46	
Respiratory system diseases or chronic lung diseases	Male	26.93	22.86	19.03	15.97	12.87	10.27	8.16	6.34	5.00	2.52
	Female	29.46	25.13	21.10	17.50	14.07	11.02	8.61	6.46	4.81	
Digestive system diseases	Male	23.82	20.38	17.02	14.16	11.53	9.25	7.32	5.75	4.54	2.07
	Female	25.89	22.14	18.67	15.53	12.63	9.99	7.71	5.84	4.61	
Skin diseases	Male	27.44	23.48	19.80	16.49	13.39	10.70	8.46	6.45	4.89	2.64
	Female	30.08	25.81	21.82	18.22	14.78	11.73	9.13	6.81	5.12	
Musculoskeletal system and connective tissue diseases	Male	25.36	21.41	17.75	14.72	11.90	9.53	7.50	5.84	4.53	−0.02
	Female	25.33	21.42	17.89	14.73	11.73	9.28	7.38	5.58	4.28	
Genitourinary system diseases	Male	26.82	22.91	19.15	15.81	12.74	10.09	7.91	6.06	4.70	3.01
	Female	29.83	25.59	21.58	17.92	14.49	11.47	8.87	6.63	5.04	

In contrast, cardiovascular diseases (CVDs) present an exception where males show slightly higher DFLE than females at older ages, with a sex gap effectively near zero or slightly negative, indicating a convergence or male advantage in years lived free of CVDs in later life. For other conditions such as respiratory and digestive diseases, skin conditions, and musculoskeletal diseases, females maintain longer DFLE at most ages, but the difference diminishes with age. Notably, musculoskeletal diseases show an almost negligible sex difference in DFLE, reflecting similar disease-free survival between sexes. Overall, these results highlight significant sex-specific disparities in healthspan across various disease domains, suggesting that women generally experience longer disease-free survival than men, but this benefit varies depending on the specific morbidity and age group.

#### 3.2.4 Age-specific contribution of morbidities in DFLE gap

**Figure 5:**
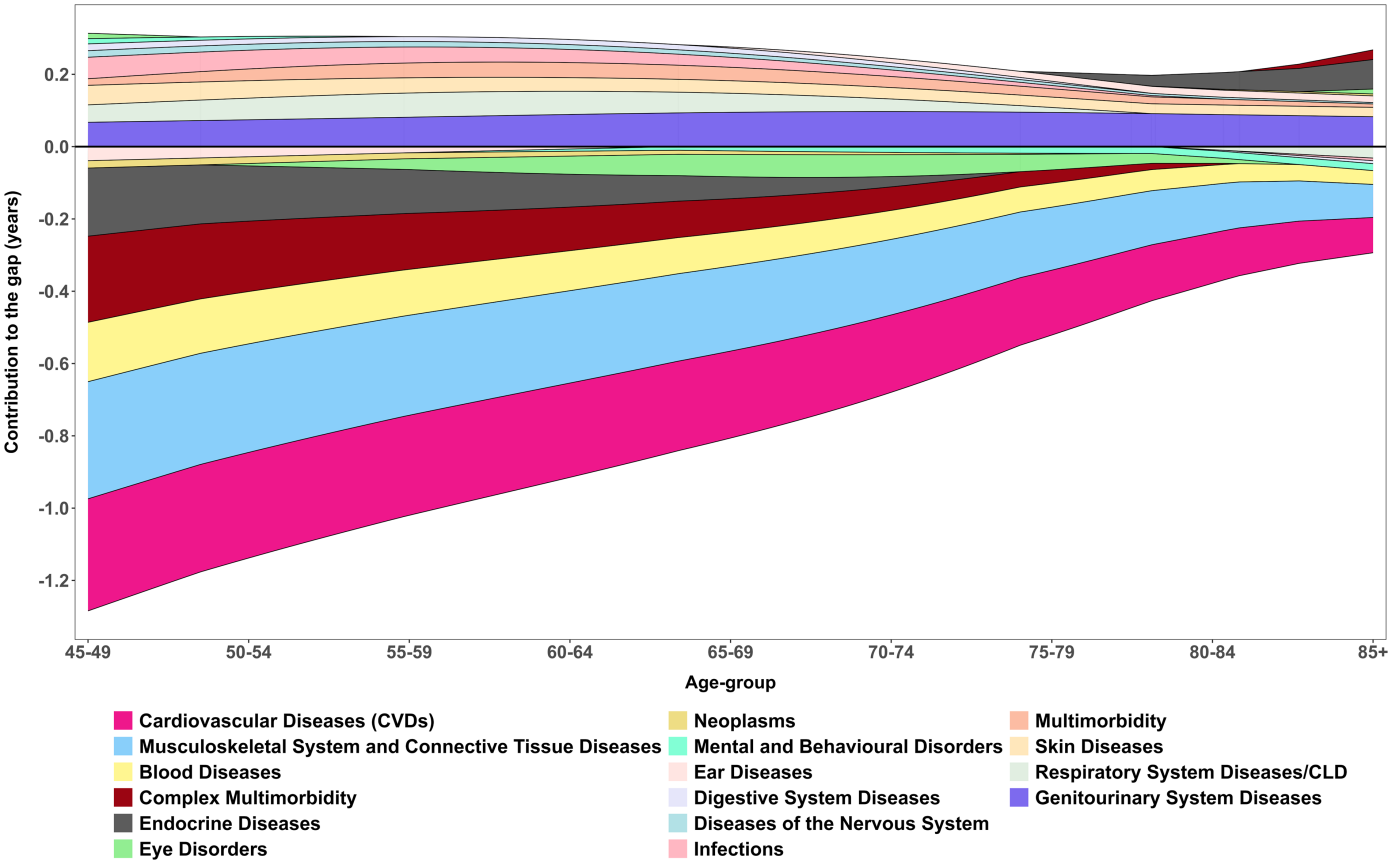
Age-specific contribution (smooth pattern) of morbidity measure to changes in DFLE inequality (gap) between male and female. These age-specific contributions sum to the total difference in DFLE between males and females, LASI Wave-1 (2017−18), India.

Figure 5 shows the age-specific smooth contributions of morbidities to the male–female gap in DFLE, with exact age-specific values presented in Table 6. CVDs consistently emerged as the strongest negative contributor across all ages, with the most pronounced effect at 45–49 years (−0.36 years). This underscores the disproportionate cardiovascular-related disability borne by women, despite their survival advantage. Musculoskeletal system and connective tissue diseases were the second largest contributor, peaking at −0.36 years between ages 70–74, highlighting the growing disability burden of non-fatal but chronic conditions in later life. Together, these patterns reaffirm the central role of NCDs in shaping sex differences in DFLE across midlife and older ages.

Respiratory system diseases/CLD, demonstrated a more nuanced effect. They contributed positively between ages 50–74 (+0.65 years overall), suggesting that advances in management and survival gains temporarily narrowed the HLE gap. However, DFLE still revealed divergence at older ages, with women maintaining only marginal advantages (e.g., 75–79 years: female 10.28 vs. male 10.77). Similarly, vision and hearing disorders showed inconsistent contributions, alternating between positive and negative values across the age spectrum. These fluctuations reflect the complex interplay of sensory decline, healthcare access, and intervention effectiveness, with vision-related improvements in older men (e.g., 80–84 years: male 66.99 vs. female 56.41) suggesting the potential role of treatment in reducing disparities.

Other conditions contributed more modestly but with distinct age-specific differences. Diabetes disproportionately reduced women’s HLE at younger ages, while infections and gastrointestinal conditions made small positive contributions, consistent with advances in treatment and control. Urogenital diseases contributed positively (+0.78 years), with benefits increasingly favouring men in older ages (e.g., 80–84 years: male 12.63 vs. female 9.40), highlighting persistent disparities in outcomes and access to care. Neurological disorders, endocrine diseases, and complex multimorbidity added smaller but non-negligible burdens, further compounding disadvantages for women at older ages. Neoplasms contributed least to sex inequality in HLE, with morbidity differences accounting for just −0.07 years, although mortality gaps were substantially larger (2.42 years).

Overall, the decomposition shows that the male–female gap in HLE is mainly driven by cardiovascular and musculoskeletal conditions, which exert the largest negative contributions across ages. In contrast, respiratory diseases, urogenital diseases, and certain sensory disorders showed positive contributions at specific ages, partially offsetting the gap. Nonetheless, it is also important to notice that among all positive contributed morbidires, Genitourinary System Diseases had the highest share in disparity.

Table 6 reveals the critical role of mortality in driving inequalities in DFLE across sexes for various morbidities and age groups. Mortality consistently contributes positively to DFLE inequality, with total mortality contributions ranging from 1.05 years for eye disorders up to 2.42 years for neoplasms. This indicates that sex differences in survival, particularly excess male mortality, substantially contribute to observed disparities in healthy lifespan.

**Table 6:** Age-specific inequality (gap) in DFLE caused by mortality and morbidity measure by sex, LASI Wave-1 (2017−18), India.

Morbidity	Measure	45–49	50–54	55–59	60–64	65–69	70–74	75–79	80–84	85+	Total
Infections	Mortality	0.34	0.27	0.41	0.38	0.36	0.31	0.21	0.11	0.00	2.37
	Morbidity	0.06	0.05	0.03	0.06	0.03	0.02	−0.01	0.00	0.00	0.25
Neoplasms	Mortality	0.35	0.27	0.42	0.39	0.37	0.31	0.21	0.11	0.00	2.42
	Morbidity	−0.01	−0.03	−0.02	−0.01	0.00	−0.03	0.00	0.03	−0.01	−0.07
Blood diseases	Mortality	0.33	0.26	0.40	0.38	0.35	0.30	0.20	0.10	0.00	2.33
	Morbidity	−0.19	−0.11	−0.10	−0.15	−0.09	−0.05	−0.06	−0.11	−0.01	−0.86
Endocrine diseases	Mortality	0.29	0.23	0.35	0.33	0.31	0.27	0.18	0.09	0.00	2.06
	Morbidity	−0.27	0.01	−0.22	−0.02	−0.09	−0.11	0.07	0.01	0.10	−0.51
Mental and behavioural disorders	Mortality	0.34	0.27	0.42	0.39	0.36	0.31	0.21	0.10	0.00	2.40
	Morbidity	0.03	−0.01	−0.01	0.02	−0.02	−0.02	−0.04	−0.01	−0.01	−0.06
Diseases of the nervous system	Mortality	0.34	0.27	0.42	0.39	0.36	0.31	0.21	0.11	0.00	2.40
	Morbidity	0.02	0.01	0.02	0.01	0.02	0.01	0.00	0.00	0.01	0.09
Eye disorders	Mortality	0.17	0.13	0.19	0.17	0.15	0.12	0.08	0.04	0.00	1.05
	Morbidity	−0.04	0.15	−0.05	−0.10	−0.02	−0.20	−0.15	0.16	0.01	−0.23
Ear diseases	Mortality	0.32	0.25	0.38	0.35	0.32	0.27	0.18	0.09	0.00	2.17
	Morbidity	−0.03	−0.06	0.01	−0.01	−0.02	0.00	0.07	0.05	−0.03	−0.01
Cardiovascular diseases (CVDs)	Mortality	0.24	0.19	0.28	0.26	0.23	0.20	0.13	0.07	0.00	1.60
	Morbidity	−0.36	−0.19	−0.25	−0.32	−0.21	−0.29	−0.22	−0.07	−0.09	−1.98
Respiratory system diseases/chronic lung diseases	Mortality	0.32	0.25	0.38	0.36	0.33	0.28	0.19	0.10	0.00	2.22
	Morbidity	−0.01	0.02	0.21	0.03	0.11	−0.02	0.01	−0.01	−0.04	0.30
Digestive system diseases	Mortality	0.29	0.22	0.34	0.32	0.30	0.26	0.17	0.09	0.00	1.98
	Morbidity	0.07	−0.06	0.00	0.01	0.06	0.04	0.01	−0.07	0.02	0.09
Skin diseases	Mortality	0.33	0.26	0.40	0.38	0.35	0.30	0.20	0.10	0.00	2.31
	Morbidity	0.04	0.11	−0.02	0.03	0.06	0.04	0.03	−0.01	0.05	0.33
Musculoskeletal system and connective tissue diseases	Mortality	0.29	0.23	0.34	0.32	0.29	0.25	0.17	0.09	0.00	1.97
	Morbidity	−0.32	−0.35	−0.21	−0.18	−0.26	−0.36	−0.13	−0.13	−0.06	−1.99
Genitourinary system diseases	Mortality	0.33	0.26	0.39	0.36	0.34	0.29	0.19	0.10	0.00	2.24
	Morbidity	0.07	0.08	0.04	0.09	0.11	0.11	0.11	0.05	0.08	0.77

Age-specific mortality contributions peak in midlife (45–64 years) across most conditions such as infections, neoplasms, blood diseases, mental and nervous system disorders, and respiratory diseases, suggesting that excess premature mortality in men during these ages significantly widens the DFLE gap. For cardiovascular diseases, mortality contributions are slightly lower but remain sizeable (total 1.60 years), further reflecting sex differences in survival related to this condition.

In later ages (75+), mortality contributions decline or reach zero, consistent with mortality selection and survivorship effects. Importantly, morbidity contributions are often negative or minimal compared to mortality, highlighting that differential disease prevalence alone does not fully explain sex-based inequalities in disease-free years. Overall, mortality differences play a dominant role in shaping sex gaps in DFLE, emphasising the need to address premature deaths among men to reduce healthy lifespan inequalities

## 4 Discussion

This is the first study that comprehensively estimated the HLE for multimorbidity, complex multimorbidity and the extensive number of morbidities for the older adult population in India. Additionally, we quantified the inequality in HLE between male–female by multimorbidity/complex multimorbidity and morbidities, attributing these disparities to prevalence rates and mortality rates. This study finally deepened the analysis by focusing on the disparity caused by complex multimorbidity. This structured discussion provides a concise synthesis of the results and highlights their broader implications for understanding sex and age-specific longevity inequalities, incorporating new insights from both mortality and morbidity factors

First, females consistently exhibited higher LE than males across all age groups and maintained slightly higher MFLE across most age groups. At 45–49, females had 25.50 multimorbidity-free years compared to 23.30 years for males and this aligns with global patterns of female longevity advantages (39, 40). At first glance of statistical view this was plausibly due to higher age-specific LE and lower multimorbidity prevalence among females (19.63%) compared to males (19.98%), which supports the idea that females not only live longer but also have relatively better health status in midlife. However, the decline in the sex gap at older ages suggests that males who survive to advanced ages may be a select, healthier subgroup (41).

Although age-specific disparities in CMFLE between male and female appear relatively small but females tend to spend significantly more years living with complex multimorbidity, and the widest differential was observed in the 45–49 age group (see Table 1; Figure 2). However, as individuals age, this sex gap gradually diminishes, indicating parity in later years. By ages 85+, the MFLE gap becomes negligible (−0.06 years), while the CMFLE gap reduces to 0.18 years. This suggests parity in health outcomes between sexes as they age for both multimorbidity and complex multimorbidity. Consistent with previous study evidence, this pattern aligns with the hypothesis that as LE increases, males and females may experience a more compressed period of morbidity, leading to a reduced sex gap in health expectancy measures (42). This persistent but reducing sex differences highlight the importance of considering age-specific and sex-specific factors in understanding the burden of multimorbidity in the mid-year of life course. This pattern persists across age groups, with females experiencing a prolonged burden of chronic conditions, while males display a sharper decline in years lived with complex multimorbidity (YLCM) as age advances.

However, a widely documented paradoxical pattern of healthy longevity by females in ageing and epidemiological studies was also observed in this study, such that females consistently spend more years as unhealthy years as complex multimorbidity but had higher age-specific LE than males (39, 43). At ages 45–49, females spend 6.09 years with multimorbidity compared to 5.85 years for males and 10.77 years with complex multimorbidity compared to 8.93 years. Subsequently, the male population also experience a sharper decline in YLCM with age, while females endure a prolonged period of life with chronic health conditions. However, this gap in YLCM between male–female showed parity in the oldest of the old age groups.

Several factors may underlie this paradox. Biologically females have a greater resilience to fatal conditions, often surviving longer despite poorer health (44). Behaviourally, females are more likely to seek healthcare and report illnesses, leading to higher diagnosis rates (45). Socially, cumulative disadvantages in nutrition, labour, and access to healthcare may exacerbate females’ late-life health burdens, yet social support networks may bolster their survival (46). In contrast, higher early-life mortality in males, often due to riskier behaviours and greater exposure to fatal diseases, results in survivor bias, where only healthier males live to older ages (47). Consequently, while females experience an expansion of morbidity, males may undergo a compression of morbidity near the end of life (19). Interestingly, the sex gap in YLCM tends to show parity in the oldest-old groups, likely due to selective survival and the equalising effects of advanced biological ageing. However, in the Indian context, long-standing sex disparities in access to education, nutrition, and health services further deepen this paradox, particularly in rural and socioeconomically disadvantaged populations (48). Additionally, patriarchal norms often deprioritise females’ health across the life course, contributing to the observed higher burden of complex multimorbidity among older Indian females. Interestingly, the sex gap in YLCM tends to also diminish in the oldest-old groups, likely due to selective survival and the equalising effects of advanced biological ageing.

Second, the decomposition analysis provides a nuanced understanding of how the age pattern of mortality and multimorbidity/complex multimorbidity contribute to HLE gaps, particularly highlighting the increasing role of complex multimorbidity over an ageing population (49–51). As outlined in research by a recent study, mortality differences largely govern disparities in HLE between sexes, with their findings revealing that such differences predominantly hinge on specific health conditions (52). However, as this study analysis articulates, the impact of complex multimorbidity becomes increasingly significant, accounting for a notable reduction in HLE gaps, specifically 0.87 years from complex multimorbidity compared to 1.47 years sourced from mortality. This shift suggests a critical balancing act in understanding health disparities, especially in older adults, where the quality of life deteriorates significantly due to the compounded effect of multiple health conditions, thus emphasising that focusing solely on mortality may undercut a comprehensive grasp of the health landscape (53).

Nonetheless, the findings underscore a critical limitation in relying predominantly on mortality-driven interpretations of health disparities. While males experience greater premature mortality, resulting in a larger HLE gap at younger ages, the role of complex multimorbidity in shaping quality of life and functional independence becomes more pronounced with ageing. For instance, in this study, mortality differences between males and females contribute 1.47 years to the healthy life expectancy gap, while complex multimorbidity burden reduces this advantage by 0.87 years, yielding a net difference of 0.60 years. This quantification demonstrates that focusing exclusively on mortality-based health metrics substantially underestimates the full scope of sex-based health inequalities in ageing populations. This aligns with prior research highlighting that females’ extended lifespan often coincides with a prolonged period of morbidity and disability, making multimorbidity a crucial determinant of later-life well-being (54). Thus, policies aimed at healthy ageing must move beyond mortality reduction and prioritise interventions that mitigate the functional burden of multimorbidity, ensuring that increased longevity translates into better health-adjusted life years rather than extended periods of ill health.

Third, chronic morbidities such as chronic bone diseases and CVDs showed the least gap in HLE between male–female, but these morbidities showed the highest inequality explained by each morbidity by −1.991 years and −1.994 years than mortality rates, because the differential of prevalence for these diseases was much significant than mortality. A recent study on sex differentials in chronic diseases among older adults in India also concluded the significant differential between males and females in the majority of chronic diseases (55). On the contrary severe disease like chronic lung diseases and cancer showed distinct pattern, such that cancer had the lowest prevalence for both male (0.48%) and female (0.77%) but showed the significant high inequality of 2.35 years in HLE out of which 2.424 years were explained by mortality and reduction of −0.073 years by the cancer prevalence. The gap in HLE was highest in chronic lung diseases (Female: 29.46, Male: 26.93, Difference: 2.53), where 2.220 years were explained by mortality and 0.65 years by morbidity. This underscores cancer’s high fatality rate, which disproportionately affects HLE disparities (56).

The results revealed nuanced findings across all 14 morbidities, showing that although women experience higher prevalence of cardiovascular, musculoskeletal, endocrine, and blood diseases, they generally enjoy longer DFLE than men, particularly at younger ages. This advantage diminishes with age, with near convergence in conditions such as cardiovascular and musculoskeletal diseases. Men, in contrast, face higher prevalence of respiratory and infectious diseases in midlife and overall shorter DFLE, reflecting faster health decline. These patterns underscore the importance of adopting sex- and age-specific health strategies, chronic disease management for women and premature morbidity prevention for men, to foster healthy ageing. One plausible explanation for the observed pattern of declining life expectancy in older age groups in India is the high mortality patterns due to severe morbidities, which can mask the true mortality pattern (57). Specifically, the presence of severe health conditions, such as those associated with multimorbidity, may be contributing to the shortening of life expectancy in older age groups rather than any underlying trend in mortality. This is particularly evident in India, where the burden of non-communicable diseases (NCDs) and other health issues is significant, especially among older adults (58). As a result, the actual mortality pattern may be obscured by the impact of these severe morbidities, leading to an apparent decline in life expectancy in older age groups. Disease-specific analyses emphasise the importance of addressing preventable and manageable conditions (e.g., CVDs, chronic bone diseases). However, the findings also highlight the need for a holistic approach that considers comorbidities and the broader determinants of health.

The focus on older females’ disproportionate burden of complex multimorbidity (see Supplementary Table S1) is critical for designing equitable healthcare policies. Sex differences in disease progression highlight the dual challenges of longevity and quality of life. Tailored interventions that address the unique needs of ageing females, including support for caregiving roles and improved access to specialised care, are essential. The findings underscore the need for targeted interventions to reduce the burden of complex multimorbidity among older females, as they experience compounded effects in later life. The study effectively captures how male–female ageing trajectories influence health outcomes, with females living longer but enduring greater multimorbidity burdens.

Finally, the data highlights the necessity and importance of prioritising chronic bone diseases, CVDs, and multimorbidity management to improve health outcomes, particularly among females. Promoting healthy ageing requires strategies aimed at reducing the prevalence and complexity of multimorbidity, especially in younger age groups, which may provide long-term benefits for females with chronic conditions.

Moreover, our study highlights significant policy implications and the need for adaptive healthcare system responses as India faces the challenges of rapid population ageing. The demonstration that mortality reduction alone provides an incomplete picture of health inequality suggests that policy frameworks must incorporate quality-of-life metrics alongside traditional mortality-based indicators. The Healthy India 2025 initiative and similar programmes should emphasise multimorbidity prevention and management as core components of healthy ageing strategies. The sex-specific patterns identified suggest the need for differentiated approaches to males’ and females’ health in ageing populations. For males, interventions should continue emphasising mortality reduction through cardiovascular disease prevention, tobacco control, and accident prevention. For females, priority should be placed on managing chronic conditions that may not be immediately life-threatening but significantly impact quality of life over extended periods. Healthcare delivery systems must be redesigned to address complex multimorbidity effectively. This includes developing integrated care models that coordinate management across multiple conditions, training healthcare providers in geriatric medicine principles, and establishing comprehensive geriatric assessment programmes. The finding that complex multimorbidity impacts are most pronounced with five or more conditions suggests that early intervention targeting individuals with 2–3 conditions may prevent progression to more complex disease patterns.

## 5 Strengths and limitations

A key strength of this study lies in its use of nationally representative data from the Longitudinal Ageing Study in India (LASI), which allows for robust, population-level insights into sex differences in life expectancy and healthy ageing. By incorporating measures of both multimorbidity-free and complex multimorbidity-free life expectancy, the analysis moves beyond simple mortality metrics to offer a more nuanced understanding of health inequalities. The study’s decomposition approach further strengthens its contribution by quantifying the relative roles of mortality and disease burden in shaping the sex gap in healthy life expectancy. Additionally, by highlighting the narrowing sex gap in advanced ages and the specific influence of chronic conditions such as cardiovascular and bone diseases, the study sheds light on critical stages in the life course where policy interventions can be most effective.

This study acknowledges several important limitations. While it attributes observed differences mainly to multimorbidity, it does not adequately examine the influence of biological, lifestyle, or socioeconomic factors that may shape sex disparities. The analysis treats sex differences in isolation, overlooking intersections with socioeconomic status, education, healthcare access, and regional variation, all of which could confound the relationship between multimorbidity and mortality. Methodologically, the use of stepwise replacement decomposition fails to account for competing risks and interactions between coexisting morbidities, potentially biasing estimates of healthy life expectancy. The disease-specific focus also neglects the mitigating role of preventive healthcare, while inconsistent contributions of conditions such as vision and hearing loss point to unexamined social and environmental determinants. Data limitations further constrain the findings. Reliance on self-reported diagnoses introduces risks of recall bias, underreporting, and misclassification, especially among older adults with low health literacy or limited disease awareness. These issues may compromise morbidity prevalence estimates and, by extension, the accuracy of healthy life expectancy calculations. Finally, the observed higher morbidity burden among women may partly reflect survival bias, since their longer life expectancy increases exposure to chronic conditions, complicating interpretations of morbidity gaps.

## 6 Conclusion

This study underscores the complex relationship between longevity and morbidity in shaping sex disparities in healthy ageing in India. While males experience higher mortality rates, females face a greater burden of years spent in complex multimorbidity, which impacts their healthy lifespan. The findings emphasise the need for public health interventions that extend beyond solely focusing on increasing life expectancy and prioritise improving the quality of life by addressing the burden of multimorbidity, especially among older females. Future research should further explore the underlying biological, socioeconomic, and lifestyle factors that contribute to these sex-based health inequalities in the ageing population of India.

## Data Availability

Publicly available datasets were analysed in this study. This data can be found at: https://www.iipsdata.ac.in/datacatalog_detail/5.

## Footnotes

1
https://posit.co/download/rstudio-desktop/
