# Supplementary material for: Outliving and outlasting: explaining sex inequities in healthy life expectancy through the lens of multimorbidity and within morbidities in older populations in India
Source: Front Public Health. 2025 Dec 2;13:1613516. doi: 10.3389/fpubh.2025.1613516 (PMC12706656; doi:10.3389/fpubh.2025.1613516)
Supplement: Supplementary file 1 [file Table_1.docx]

**Supplementary Table S1: ICD-10 Classification of thirty-six diseases and conditions retrieved from the LASI Wave-1 (2017−18), India**

| **Sl. No** | **ICD-10 Chapter Classification** | **Included Diseases / Disorders / Conditions with ICD-10 Codes** |
| --- | --- | --- |
| **I** | **Infections** | Tuberculosis (A15–A19), Hepatitis/Jaundice (B15–B19) |
| **II** | **Neoplasms** | Cancer/Malignant Neoplasms (C00–C97) |
| **III** | **Blood Diseases** | Anaemia (D50–D64) |
| **IV** | **Endocrine Diseases** | Diabetes Mellitus (E10–E14), Thyroid (E00–E07), High Cholesterol (E78) |
| **V** | **Mental and Behavioural Disorders** | Depression (F32–F33), Dementia (F01–F03), Psychiatric Conditions (F00–F99) |
| **VI** | **Diseases of the Nervous System** | Neurological Conditions (G00–G99) |
| **VII** | **Eye Disorders** | Cataract (H25–H26), Glaucoma (H40–H42), Hypermetropia (H52.0), Myopia (H52.1), Presbyopia (H52.4) |
| **VIII** | **Ear Diseases** | Hearing Conditions (H90–H91) |
| **IX** | **Cardiovascular Diseases (CVDs)** | Hypertension (I10–I15), Heart Attack (I21), Heart Blockage (I20–I25), Heart Failure (I50), Arrhythmias Heart/Cardiac arrhythmias (I49), Rheumatic Heart (I01–I09) Heart Congenital/Structural Disorders (Q24.8 - 9), Stroke (I63–I64) |
| **X** | **Respiratory System Diseases/Chronic Lung Diseases (CLD)** | Asthma (J45), Bronchitis (J20), Chronic Obstructive Pulmonary Disease (COPD) (J44) |
| **XI** | **Digestive System Diseases** | Gastrointestinal Conditions (K00–K93) |
| **XII** | **Skin Diseases** | Skin Conditions (L00–L99) |
| **XIII** | **Musculoskeletal System and Connective Tissue Diseases** | Arthritis (M13), Rheumatism (M79), Osteoporosis (M80–M82) |
| **XIV** | **Genitourinary System Diseases** | Urogenital Conditions (N00–N99) |

**Supplementary Table S2: Age-specific healthy life expectancy (HLE) of multimorbidity and complex multimorbidity stratified by sex, LASI Wave-1 (2017−18), India**

| **Age group** | | | **45-49** | **50-54** | **55-59** | **60-64** | **65-69** | **70-74** | **75-79** | **80-84** | **85+** |
| --- | --- | --- | --- | --- | --- | --- | --- | --- | --- | --- | --- |
| **Multimorbidity** | **Two** | **Male** | 23.3 | 19.84 | 16.71 | 14.01 | 11.39 | 9.15 | 7.24 | 5.57 | 4.38 |
|  |  | **Female** | 25.5 | 21.88 | 18.5 | 15.4 | 12.47 | 9.98 | 7.72 | 5.96 | 4.32 |
| **Complex Multimorbidity** | **Three** | **Male** | 25.35 | 21.53 | 17.95 | 14.89 | 12.03 | 9.57 | 7.42 | 5.51 | 4.22 |
|  |  | **Female** | 27.3 | 23.37 | 19.68 | 16.33 | 13.18 | 10.37 | 7.89 | 5.82 | 4.42 |
|  | **Four** | **Male** | 26.55 | 22.55 | 18.87 | 15.75 | 12.7 | 10.16 | 7.98 | 6.18 | 4.83 |
|  |  | **Female** | 28.71 | 24.48 | 20.61 | 17.11 | 13.8 | 11.07 | 8.61 | 6.46 | 4.89 |
|  | **Five** | **Male** | 27.92 | 23.82 | 19.93 | 16.53 | 13.36 | 10.7 | 8.49 | 6.55 | 5.2 |
|  |  | **Female** | 30 | 25.67 | 21.64 | 17.96 | 14.51 | 11.49 | 9.03 | 6.81 | 5.04 |
|  | **Six** | **Male** | 28.54 | 24.38 | 20.45 | 17 | 13.78 | 10.98 | 8.7 | 6.7 | 5.18 |
|  |  | **Female** | 30.66 | 26.27 | 22.16 | 18.39 | 14.86 | 11.81 | 9.19 | 6.89 | 5.24 |
|  | **Seven** | **Male** | 28.78 | 24.63 | 20.67 | 17.21 | 13.96 | 11.17 | 8.84 | 6.78 | 5.32 |
|  |  | **Female** | 31.13 | 26.7 | 22.55 | 18.78 | 15.22 | 12.09 | 9.42 | 7.09 | 5.33 |
|  | **Eight Plus** | **Male** | 28.83 | 24.7 | 20.75 | 17.26 | 14.02 | 11.21 | 8.85 | 6.81 | 5.28 |
|  |  | **Female** | 30.98 | 26.54 | 22.37 | 18.61 | 15.03 | 11.85 | 9.37 | 7.07 | 5.3 |
